# Comparative Analysis of Traditional and Modern Fermentation for Xuecai and Correlations Between Volatile Flavor Compounds and Bacterial Community

**DOI:** 10.3389/fmicb.2021.631054

**Published:** 2021-04-29

**Authors:** Jianming Zhang, Chengcheng Zhang, Xiaoting Xin, Daqun Liu, Wenwu Zhang

**Affiliations:** ^1^Institute of Food Science, Zhejiang Academy of Agricultural Sciences, Hangzhou, China; ^2^Hangzhou Trendbiotech Co., Ltd, Hangzhou, China

**Keywords:** flavor compounds, bacterial community, Xuecai, traditional fermentation, modern fermentation

## Abstract

Differences in flavor compounds and bacterial communities of Xuecai by traditional and modern fermentation are poorly understood. Allyl isothiocyanate (E9), ethyl acetate (E1), 3-butenenitrile (N1), phenol (P1), ethanol (A1), and 3-(2,6,6-trimethyl-1-cyclohexen-1-yl) acrylaldehyde (L11) were the main flavor compounds that differed between Xuecai produced by traditional and modern fermentation. Among these compounds, the contents of N1 and E9 were higher in modern fermentation Xuecai. Traditional fermentation Xuecai possessed higher contents of A1, P1, E1, and L11. High-throughput sequencing showed that *Lactobacillus-*related genera was the most abundant genus (50%) in modern fermentation Xuecai. However, in traditional fermentation Xuecai, *Halanaerobium* (29.06%) and *Halomonas* (12.96%) were the dominant genera. Halophilic bacteria (HB) positively contribute to the flavor of Xuecai. Carbohydrate metabolism and amino acid metabolism were the most abundant pathways associated with the bacterial communities of the Xuecai. This indicated that Xuecai flavor formation is mainly dependent on protein and carbohydrate degradation. This study provides a novel insight that HB may be important for flavor formation of Xuecai.

## Introduction

Fermented vegetables, such as Suancai, Paocai, Jiangshui, and Zhacai, are very popular in China (Liang et al., 2018; Liu et al., 2019a, b; Xiao et al., 2020). In recent years, fermented vegetables have aroused an increasing amount of attention due to their pleasant taste, nutritional value, and health benefits such as reducing the risks of metabolic disorders and age-related diseases (Sebastian et al., 2020). Potherb mustard (*Brassica juncea* var. crispifolia) is a member of the Cruciferae family, and its leaves are generally pickled (Fang et al., 2008). Pickled potherb mustard is often called “Xuecai” in China and is usually cooked with meats, fish, or other dishes (Zhao et al., 2007). Xuecai is extensively consumed in China, especially in the Hangzhou Bay area. Every year, millions of tons of potherb mustard are processed into Xuecai by curing in Jiaxing and Ningbo, Zhejiang Province. Therefore, Xuecai from Jiaxing and Ningbo is recognized as a national geographic agricultural product in China.

However, the method used for pickling Xuecai differs between Jiaxing and Ningbo. Xuecai from Jiaxing is pickled using traditional technology. In general, potherb mustard is prepared at the beginning of every winter or at the beginning of every spring. After harvesting, the potherb mustard leaves are washed and drained, wilted in the sun, and then mixed with sodium chloride (NaCl) at a concentration of 4–5% (w/w). The potherb mustard leaves are kneaded with salt and compacted with stones. The next day, the pickled potherb mustard leaves are transferred to a new container and are kneaded again with 2–3% (w/w) NaCl to form juice. After marinating for 2 days, the pickled potherb mustard leaves are packed into earthenware pots in layers. The earthenware pots are inverted for 12 h in order to drain them of the brine. To end the procedure, the earthenware pots are sealed using rice chaff mud and are kept in a cool and well-ventilated place for fermentation (Zhao et al., 2007; Zhang et al., 2021).

The technology used to Xuecai in Ningbo is more convenient than that used in Jiaxing and is considered a modern fermentation technology. In brief, after harvesting, potherb mustard leaves are directly packed into cement containers, and a salt concentration of 10–15% (w/w) is added. The containers are sealed using plastic film. Thus, these differences in the fermentation technology and curing environment between Jiaxing and Ningbo affect the flavor of the Xuecai. In fact, to date, little information has been reported on the volatile flavor compounds in Xuecai produced using the different fermentation technologies.

The bacterial communities in fermented vegetables play a vital role in the formation of flavor compounds (Huang et al., 2018; Chen et al., 2019). The metabolic activities performed by the bacteria, such as the degradation of proteins and carbohydrates, are important for producing flavor compounds during fermentation (Wang et al., 2020). These flavor compounds provide the sensory characteristics of fermented vegetables. Previous studies have found that lactic acid bacteria (LAB), including *Lactobacillus*-related genera (*Lactobacillus*, *Paucilactobacillus*, *Lacticaseibacillus*, etc.) (Zheng et al., 2020; Kim et al., 2021), *Pediococcus* and *Lactococcus*, are the main bacteria responsible for the flavor of fermented vegetables (Liu et al., 2018, 2019a; Guan et al., 2020). However, the dominant bacterial flora associated with fermented vegetables subjected to different traditional fermentation methods differ greatly due to differences in raw materials, geography, climate, and manufacturing processes. Until now, the network correlations between functional bacteria and flavor compounds in Xuecai remain poorly characterized (Zhang et al., 2019, 2021). In this work, headspace solid-phase microextraction (HS-SPME) combined with gas chromatography-mass spectrometry (GC-MS), was applied to determine the types and contents of flavor compounds associated with Xuecai that has been produced by traditional and modern fermentation technologies. High-throughput sequencing (HTS) was applied to reveal the major bacterial community members. The potential correlations between the bacterial communities (and their associated metabolic pathways) and flavor compounds in the different types of Xuecai were revealed through correlations networks and Kyoto Encyclopedia of Genes and Genomes (KEGG) pathway analysis. The aim of this work was to evaluate and compare the flavor compounds of Xuecai produced using traditional and modern fermentation technologies, and to facilitate further exploration of bacterial resources in Xuecai.

## Materials and Methods

### Sample Collection

Four samples of Xuecai that had been produced using traditional fermentation technology (JX) were collected from Jiaxing, Zhejiang Province in China at December 2019. Five samples of Xuecai that had been produced using modern fermentation technology (NB) were collected from Ningbo, Zhejiang Province in China at December 2019. These samples were made from different factories or homemade. The sample information was shown in Table 1 and Supplementary Figure 1. The technology for traditional fermentation and modern fermentation were described in detail at introduction.

**TABLE 1:** Physicochemical properties between traditional fermentation Xuecai and modern fermentation Xuecai.

Order	Group	Region	Code	Source	Fermentation (month)	Titratable acidity (TA, g/100 g)	pH	Salt content (%)	Nitrite concentrations (mg/kg)
1	Traditional fermentation Xuecai (JX)	Jiashan, Jiaxing	JX-1	Homemade1	8	2.30	3.77	8.50	1.58
2		Jianshan, Jiaxing	JX-2	Homemade2	8	1.96	3.75	8.65	0.69
3		Jianshan, Jiaxing	JX-3	Factory1	8	1.53	3.79	7.60	0.69
4		Haining, Jiaxing	JX-4	Factory2	10	1.42	4.21	9.60	1.77
	Average of JX				8.5	1.75	3.88	8.59	1.18
5	Modern fermentation Xuecai (NB)	Yinzhou, Ningbo	NB-1	Factory3	9	0.82	4.90	13.90	1.39
6		Yinzhou, Ningbo	NB-2	Factory4	9	0.64	5.20	14.70	0.94
7		Yinzhou, Ningbo	NB-3	Factory5	7	0.48	5.49	10.60	1.20
8		Cixi, Ningbo	NB-4	Factory6	7	1.84	4.10	11.90	1.26
9		Cixi, Ningbo	NB-5	Factory7	8	1.64	4.18	12.90	0.50
	Average of NB				8	1.08	4.77	12.80	1.06

*The raw material of all samples was Potherb mustard (Brassica juncea var. crispifolia). The raw material of JX1, JX2, and JX3 were planted in Jiashan county, Jiaxing City. The raw material of JX4 was planted in Haining county, Jiaxing City. The raw material of NB1, NB2, and NB3 were planted in Yinzhou county, Ningbo City. The raw material of NB4 and NB5 were planted in Cixi country, Ningbo City. Hamemade1 and Hanemade2 were two different farmhouses. Factory1, Donglinhu vegetable factory; Factory2, Yunlou Food Co., Ltd.; Factory3, Yinfa green Food Co., Ltd.; Factory4, Sanfenkewei Food Co., Ltd.; Factory5, Ziyuntang Aquatic Food Co., Ltd.; Factory6, Shenqi Vegetable Co., Ltd.; Factory7, Yufeng Food Co., Ltd.*

### Analysis of Physicochemical Properties

Xuecai were homogenized in a pulper. The slurry was filtered through the gauze to obtain juice. Juice was used to analysis of physicochemical properties. The pH and salt content of all the Xuecai samples were measured using a pH meter (METTLER TOLEDO, Zurich, Switzerland) and a salinity meter (ATAGO, Tokyo, Japan), respectively. The titratable acidity (TA) and the nitrite content were analyzed using the titration method described by the Chinese national standard (GB/T5009.235-2016, 2016; Gb/T5009.33-2016, 2016; Wu et al., 2015; Chuah et al., 2016).

### HS-SPME GCMS Analysis

Juice was obtained from the Xuecai samples for volatile analysis. A volume of the juice (3 g) was pipetted into a vial and 10 μL of 3-octanol solution (32.75 mg/L; internal standard) was added. The vials were sealed tightly. The headspace (HS) volatiles were extracted by exposing the Xuecai juice to SPME fiber composed of 50/30 μm carboxen/divinylbenzene/polydimethylsiloxane (CAR/DVB/PDMS; ANPEL Laboratory Technologies Inc., Shanghai, China) at 50°C. The fiber was exposed to the headspace of the capped vial to adsorb volatile substances for 30 min. The fiber was then withdrawn and introduced into a heated injection port of the gas chromatography-mass spectrometer.

A GC-MS system (Agilent Technologies, Palo Alto, CA, United States) equipped with an HP-5 MS column (30 m × 250 μm × 0.25 μm; Agilent Technologies, Palo Alto, CA, United States) was used for analysis. The volatiles were desorbed from the SPME fiber at 250°C in the injection port. The GC temperature was programmed as follows: held at 35°C for 5 min, increased to 150°C at 5°C/min and held for 3 min, increased to 190°C at 8°C/min and held for 1 min, and increased to 250°C at 30°C/min and held for 5 min. Both the ion source and MS transfer line temperatures were maintained at 250°C. Helium was used as the carrier gas with a flow rate of 1 mL/min, and the injection was performed in splitless mode. The mass spectrometer was operated in electron ionization (EI) mode at 70 eV with a scan range from m/z 35 to 400 (Shi et al., 2020).

### Qualitative and Quantitative Analysis

The data collected from the GC-MS were processed using Agilent MassHunter Qualitative Analysis software. Volatile compounds were identified according to the NIST MS Search 2.0 database. The retention indexes (RIs) of the compounds were calculated from a series of n-alkanes (C7–C40; Sigma, St. Louis, MO, United States), which were subjected to the same GC-MS analysis program as the sample. The relative content of each volatile compound (μg/kg) was quantified by calculating the ratio of the peak area of each volatile compound to the peak area of the internal standard (3-octanol, Aladdin, Shanghai, China) (Supplementary Figure 2; Wang et al., 2020).

(1) $$Concentration\left(\frac{\mu g}{kg}\right)=\frac{P\times 0.3275\mu g\left(3-octanol\right)}{M}\times 1000$$

where, *P* is the ratio of the peak area of each volatile compound to the peak area of internal standard, and *M* is the weight of the Xuecai juice (g).

### High-Throughput Sequencing Analysis

Microorganisms were enriched by centrifugation (5000 g, 5 min) from Xuecai. Then, bacterial community genomic DNA was extracted using a DNA Kit (Omega Bio-tek, Norcross, GA, United States) according to the manufacturer’s instructions. The hypervariable V3–V4 region of the bacterial 16S rRNA gene was amplified using the primer pair 338F and 806R. The purified PCR products were sequenced using an Illumina MiSeq platform (Illumina, San Diego, CA, United States) according to the method described by Huang et al. (2020).

### Statistical Analyses

All the results are presented as the averages of three replicates. A supervised pattern recognition method (orthogonal projection on latent structure-discriminant analysis; OPLS-DA) was performed to analyze the volatile compounds in the Xuecai samples using SIMCA software (ver. 14.1; UMETRICS, Sweden). The linear discriminant analysis (LDA) effect size (LEfSe) algorithm was carried out to analyze the different bacterial taxa on the online Galaxy platform^1^ (Goecks et al., 2010). Spearman’s correlation coefficients (*r*) were calculated using SPSS software (ver. 19.0; SPSS Inc., United States), in which | *r*| > 0.7 with *P* < 0.05 was considered to be a robust correlation (Huang et al., 2019). The correlation networks between bacterial communities and flavor compounds were visualized *via* Cytoscape software (ver. 3.6.1). Predictive functional genomic analysis of the bacterial communities was carried out using Phylogenetic Investigation of Communities by Reconstruction of Unobserved States (PICRUSt) 1.0.0 based on the Greengene 16S rRNA gene dataset. Statistical significance was acknowledged when P-values were below 0.05 (^∗^*P* < 0.05, ^∗∗^*P* < 0.01). The graph presentations were generated using Origin Software (ver. 8.6; OriginLab Corporation, Hampton, MA, United States).

## Results and Discussion

### Comparing Physicochemical Properties of Xuecai Produced by Traditional and Modern Fermentation

As expected, the salt content and pH were much lower in the traditional fermentation Xuecai (8.59% and 3.88) than in the modern fermentation Xuecai (12.80% and 4.77). The TA was higher in the traditional fermentation Xuecai (1.75/100 g) than in the modern fermentation Xuecai (1.08/100 g; Table 1). The TA of mature fermented vegetables has been shown to generally be greater than 0.3/100 g (Liu et al., 2019a). Thus, these two types of Xuecai had reached maturity. In addition, the nitrite content in the traditional and modern fermentation Xuecai was far lower than the safety limit for nitrite content (20 mg/kg, Table 1) described in the Chinese National Food Standard (GB 2762-2017). This suggests that all of the Xuecai sampled in this study were safe for consumers.

### Identification of Volatile Flavor Compounds

**FIGURE 1:**
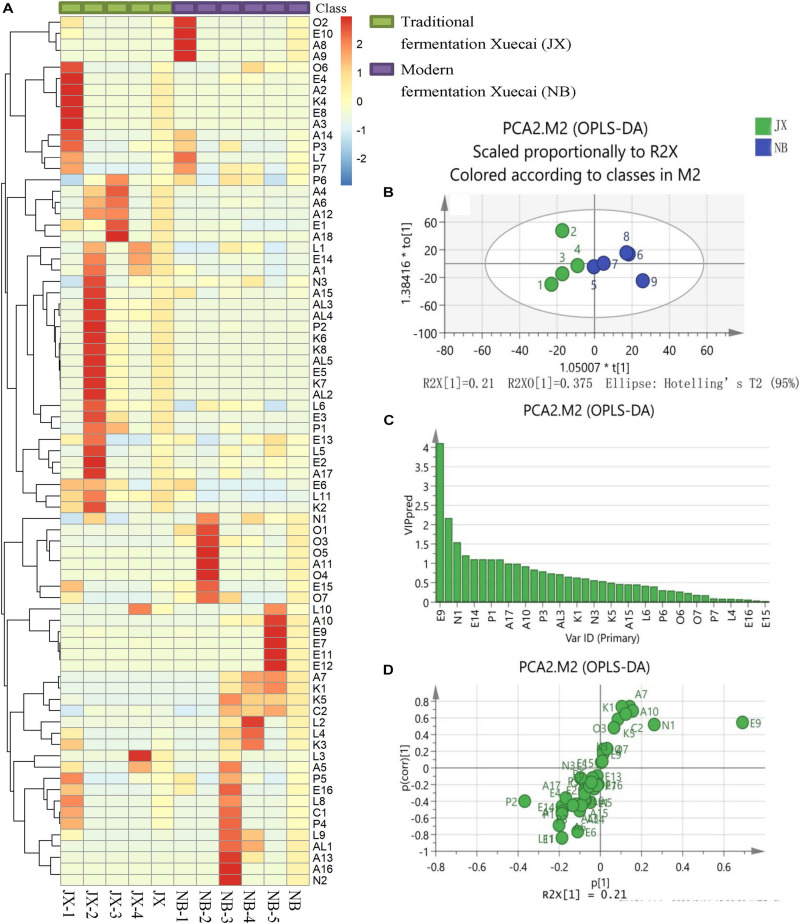
Heatmap visualization **(A)**, OPLS-DA score plot **(B)**, VIP plot **(C)**, and loading S-plots **(D)** of the flavor compounds in traditional fermentation Xuecai and modern fermentation Xuecai based on GCMS data. For interpretation of the code in this figure, the reader is referred to the Table 2 and Supplementary Table 2.

Changes in flavor compounds due to microbial metabolism are thought to contribute to these changes in physicochemical properties. Therefore, the volatile flavor compounds and bacterial flora were further compared in the traditional and modern fermentation Xuecai using HS-SPME GC-MS and HTS in order to reveal correlations between volatile flavor profiles and bacterial community profiles. The identification of volatile flavor compounds was based on the retention time and RI (Table 2), as obtained from GC-MS (Supplementary Figure 2). In total, 77 volatile compounds were detected, including 16 esters, 11 aldehydes, 18 alcohols, 8 ketones, 2 acids, 3 nitriles, 5 alkenes, 7 phenols, and 7 other compounds (Figure 1A and Table 2). An average of 30 types of volatile compound existed in each sample (Supplementary Table 1). There were 15 compounds (E3, A2, A3, A4, A12, A18, K2, K4, K6, K7, K8, AL2, AL3, AL4, and AL5) that only existed in JX samples and 16 compounds (E11, E12, L2, A7, A8, A9, A10, A11, A13, A16, K1, N2, AL1, O1, O4, and O5) that only existed in NB samples (Supplementary Table 2). For Xuecai from JX, the main types of volatile compounds were alcohols (25%), esters (21%), nitriles (17%), and phenols (17%), which contributed to 80% of the total volatiles content (Supplementary Table 2). However, in Xuecai from NB, there was a higher content of nitriles, which constituted 33% of the total volatiles, followed by esters (27%) and alcohols (16%). These results suggest that the volatile composition differed according to the production area, which may be related to the manufacturing processes and environmental conditions such as temperature, humidity, salinity, and microorganisms.

**TABLE 2:** Average contents of esters, aldehydes, alcohols, ketones, acids, nitriles, alkenes, phenols, others, and their distribution ranges (in parenthesis) in traditional fermentation Xuecai (JX), and modern fermentation Xuecai (NB).

Code^*a*^	CAS No.	Compounds	RT^*b*^	RI^*c*^	Content of JX (μg/kg)	Content of NB (μg/kg)
**Esters**						
E1	141-78-6	Ethyl acetate	2.14	621	36.32 (8.46–39.31)	5.46 (0–10.24)
E2	109-60-4	Propyl acetate	3.71	708	20.06 (0–78.11)	5.28 (0–9.03)
E3	623-52-7	Methyl butyrate	3.90	724	5.50 (0–15.53)	nd
E4	97-62-1	Ethyl isobutyrate	4.80	732	30.25 (0–121.00)	5.07 (0–13.40)
E5	105-54-4	Ethyl butyrate	6.61	792	15.78 (0–53.69)	nd
E6	123-86-4	Butyl acetate	7.16	808	8.30 (0–12.86)	2.12 (0–10.62)
E7	56601-42-4	Cyclopropyl isothiocyanate	9.48	868	1.51 (0–6.02)	38.87 (0–194.35)
E8	123-92-2	Isoamyl acetate	9.62	872	0.47 (0–1.87)	nd
E9	057-06-7	Allyl isothiocyanate	9.83	878	28.68 (0–68.19)	314.97 (26.43–1399.05)
E10	591-81-1	4-hydroxybutyric acid	11.54	923	0.34 (0–1.35)	1.84 (0–9.18)
E11	592-82-5	Butyl isothiocyanate	11.60	925	nd	6.70 (0–33.50)
E12	591-82-2	Isobutyl isothiocyanate	12.40	947	nd	3.34 (0–16.68)
E13	10348-47-7	Ethyl 2-hydroxy-4-methylvalerate	16.16	1055	6.04 (0–16.62)	5.18 (0–9.91)
E14	108-84-9	1,3-dimethylbutyl acetate	19.25	1162	93.43 (41.73–149.89)	39.63 (29.37–46.99)
E15	103-45-7	Phenethyl acetate	22.21	1252	0.96 (0–3.85)	1.48 (0–5.89)
E16	628-97-7	Palmitic acid ethyl ester	38.82	1987	0.44 (0–1.75)	0.74 (0–2.75)
**Aldehydes**						
L1	110-62-3	Valeraldehyde	3.26	696	6.18 (1.72–9.71)	2.08 (0–5.83)
L2	66-25-1	Hexanal	6.44	787	nd	1.83 (0–6.77)
L3	098-01-1	Furfural	7.66	821	6.06 (0–24.25)	1.14 (0–5.72)
L4	3268-49-3	3-(methylthio)propionaldehyde	10.65	899	0.32 (0–1.28)	1.10 (0–3.42)
L5	100-52-7	Benzaldehyde	12.65	954	49.80 (22.59–122.25)	38.97 (22.21–50.56)
L6	122-78-1	Phenylacetaldehyde	15.60	1038	15.42 (7.16–33.01)	7.21 (0–14.53)
L7	124-19-6	1-nonanal	17.63	1099	0.74 (0–2.955)	0.73 (0–3.65)
L8	5779-94-2	2,5-dimethylbenzaldehyde	21.00	1210	0.82 (0–3.26)	0.64 (0–3.18)
L9	432-25-7	β-cyclocitral	21.18	1216	0.42 (0–1.68)	1.91 (0–5.82)
L10	4411-89-6	2-phenyl-2-butenal	22.66	1268	4.74 (0–18.96)	3.64 (0–18.19)
L11	4951-40-0	3-(2,6,6-trimethyl-1-cyclohexen-1-yl) acrylaldehyde	26.09	1395	24.22 (13.86–41.29)	3.81 (0–14.25)
**Alcohols**						
A1	64-17-5	Ethanol	1.40	482	65.00 (26.43–116.62)	24.40 (12.95–42.08)
A2	71-23-8	1-propanol	1.77	547	0.84 (0–3.36)	nd
A3	78-92-2	2-butanol	2.04	597	0.49 (0–1.96)	nd
A4	71-36-3	1-butanol	2.79	657	89.55 (0–129.25)	nd
A5	123-51-3	3-methyl-1-butano	4.31	716	3.35 (0–8.22)	2.08 (0–10.39)
A6	71-41-0	1-pentanol	5.40	752	13.37 (0–30.53)	1.37 (0–3.52)
A7	1576-95-0	Cis-2-penten-1-ol	5.50	755	nd	11.85 (0–24.88)
A8	565-60-6	3-methyl-2-pentanol	5.84	767	nd	0.85 (0–4.27)
A9	544-12-7	Trans-3-hexen-1-ol	8.69	848	nd	0.89 (0–4.46)
A10	928-96-1	Cis-3-hexen-1-ol	8.75	850	nd	13.51 (0–45.10)
A11	98-00-0	Furfuryl alcohol	9.06	858	nd	1.30 (0–6.52)
A12	111-27-3	1-hexanol	9.43	867	8.93 (0–17.24)	nd
A13	505-10-2	3-methylthiopropanol	13.47	976	nd	1.13 (0–4.30)
A14	104-76-7	2-ethylhexanol	15.22	1026	1.85 (0–7.40)	0.86 (0–4.31)
A15	98-85-1	Dl-1-phenethylalcohol	16.32	1060	17.66 (6.83–44.34)	1.18 (6.63–20.54)
A16	5337-72-4	2,6-dimethylcyclohexanol	17.71	1102	nd	1.21 (0–6.05)
A17	060-12-8	Phenethyl alcohol	18.00	1112	113.09 (57.01–245.17)	77.97 (56.19–100.38)
A18	122-97-4	3-phenyl-1-propanol	21.50	1227	14.70 (0–58.79)	nd
**Ketones**						
K1	1629-58-9	Ethyl vinyl ketone	3.04	678	nd	6.83 (0–14.67)
K2	NA	Oxime-, methoxy-phenyl	11.89	933	20.98 (0–56.97)	nd
K3	110-93-1	6-methyl-5-hepten-2-one	13.72	983	1.14 (0–4.54)	2.07 (0–6.78)
K4	3879-26-3	Nerylacetone	27.39	1446	0.23 (0–0.91)	nd
K5	79-77-6	β-lonone	28.28	1481	0.28 (0–1.13)	7.11 (0–14.80)
K6	532-65-0	Ar-turmerone	33.53	1663	24.11 (0–80.35)	nd
K7	180315-67-7	Tumerone	33.63	1666	8.06 (0–27.21)	nd
K8	87440-60-6	Curlone	34.38	1696	6.50 (0–21.49)	nd
**Acids**						
C1	124-07-2	Octanoic acid	20.19	1183	1.12 (0–4.49)	1.18 (0–5.88)
C2	142-62-1	Hexanoic acid	14.51	1005	7.35 (2.32–9.39)	17.96 (3.20–29.47)
Nitriles						
N1	109-75-1	3-butenenitrile	2.67	658	59.53 (8.61–2155.92)	109.27 (45.31–210.63)
N2	140-29-4	Benzeneacetonitrile	18.71	1135	nd	0.75 (0–3.76)
N3	645-59-0	3-phenylpropionitrile	21.76	1237	234.41 (0–636.58)	172.53 (79.83–295.47)
**Alkenes**						
AL1	26456-76-8	3,5,5-trimethyl-2-hexene	13.28	971	nd	1.81 (0–5.18)
AL2	644-30-4	α-curcumene	28.20	1477	65.81 (0–222.89)	nd
AL3	495-60-3	α-zingiberene	28.50	1489	20.99 (0–67.92)	nd
AL4	495-61-4	β-bisabolene	28.87	1503	11.62 (0–35.75)	nd
AL5	20307-83-9	β-sesquiphellandrene	29.29	1516	35.10 (0–120.69)	nd
**Phenols**						
P1	108-95-2	Phenol	14.00	990	39.21 (0–86.97)	3.30 (0–16.48)
P2	106-44-5	4-hydroxytoluene	17.06	1082	205.41 (4.90–744.13)	7.55 (0–25.34)
P3	123-07-9	4-ethylphenol	19.79	1170	20.85 (0–69.97)	14.62 (0–44.26)
P4	1195-09-1	2-methoxy-5-methylphenol	20.38	1189	0.43 (0–1.73)	0.48 (0–2.41)
P5	2785-89-9	4-ethyl-2-methoxyphenol	22.85	1275	12.95 (0–42.96)	13.51 (0–36.36)
P6	7786-61-0	4-hydroxy-3-methoxystyrene	23.81	1310	22.52 (17.81–46.83)	22.06 (5.63–34.50)
P7	96-76-4	2,4-di-tert-butylphenol	28.99	1507	0.96 (0–3.84)	1.53 (0–3.96)
**Others**						
O1	624-92-0	Dimethyl disulfide	4.20	712	nd	7.19 (0–24.91)
O2	106-42-3	P-xylene	9.13	860	0.35 (0–1.38)	0.75 (0–3.76)
O3	111-76-2	2-Butoxyethanol	10.75	902	0.05 (0–0.18)	5.68 (0–17.09)
O4	3658-80-8	Dimethyl trisulfide	14.12	993	nd	3.95 (0–19.77)
O5	3238-55-9	2-Propionylpyridine	18.25	1120	nd	2.43 (0–12.15)
O6	496-16-2	2,3-Dihydrobenzofuran	21.39	1224	14.59 (0–58.37)	9.03 (0–30.29)
O7	1014-60-4	1,3-di-tert-butylbenzene	22.09	1248	1.42 (0–5.67)	5.34 (0–14.04)

*^a^Compound codes. ^b^Retention time (min). ^c^Retention index. nd, not detected; NA, not applicable.*

### Volatile Flavor Compound Composition

Sixteen ester compounds were identified, which contributed 21 and 27% of the total volatiles content in JX and NB, respectively. Methyl amyl acetate (E14) was the most abundant ester in JX samples, followed by ethyl acetate (E1), ethyl isobutyrate (E4), and allyl isothiocyanate (E9). However, E9 was the most abundant ester in NB samples, followed by E14 and cyclopropyl isothiocyanate (E7). Ester compounds, especially isothiocyanates, which are characterized by pungency and hot-like odors, are considered to be major flavor components of fermented vegetables (Zhang et al., 2019). For example, Buttery et al. (1976) reported that phenylethyl isothiocyanate was an important component of cabbage aroma. In the present study, four isothiocyanates (E7, E9, E11, and E12) were detected in Xuecai. Among them, the content of allyl isothiocyanate (E9) was high in JX and NB samples, which demonstrated that allyl isothiocyanate may make a large contribution to the flavor of Xuecai.

3-Butenenitrile (N1), benzeneacetonitrile (N2), and 3-phenylpropionitrile (N3) were detected in the Xuecai using GC-MS, and in combination they contributed 17 and 34% of the total volatiles content in JX and NB samples, respectively. Previous reports have demonstrated that nitriles are also the main volatile flavor components in Cruciferae products such as pickled mustard tuber (Liu et al., 2010). This can be explained by the fact that vegetables of the Cruciferae family have high contents of glucosinolates, which can be degraded into nitriles and isothiocyanates *via* the action of myrosinase during fermentation. The present results showed that the contents of 3-butenenitrile (N1) and 3-phenylpropionitrile (N3) were very high in the Xuecai. These two compounds have also been reported to be abundant in other fermented products (Zhao et al., 2007; Ishihara et al., 2018).

Besides esters and nitriles, alcohols are common and important flavor components in some vegetables. In the present study, a total of 18 alcohols were identified, which contributed 25 and 16% of the total volatiles content in JX and NB samples, respectively. Among them, phenethyl alcohol (A17) was the most abundant alcohol both in JX and NB samples; phenethyl alcohol can provide a floral and rosy odor (Zhao et al., 2007). However, 1-propanol (A2), 2-butanol (A3), 1-butanol (A4), 1-hexanol (A12), and 3-phenyl-1-propanol (A18) were only detected in JX samples. Cis-2-penten-1-ol (A7), 3-methyl-2-pentanol (A8), trans-3-hexen-1-ol (A9), cis-3-hexen-1-ol (A10), furfuryl alcohol (A11), 3-methylthiopropanol (A13), and 2,6-dimethylcyclohexanol (A16) were only detected in NB samples. These differences in the types of alcohols present indicated that the bacterial communities of the JX and NB samples were largely different. This is because the synthesis of alcohols is mainly determined by bacterial fermentation.

Seven phenol compounds were identified in the Xuecai samples. Notably, phenol compounds contributed 17% of the total volatiles content in JX samples, which was far higher than the phenol content in NB samples (8%). In particular, the average content of 4-hydroxytoluene (P2) reached 205.41 μg/kg in JX samples. According to previous reports, phenols are responsible for smoky and phenolic odors (Liu et al., 2020). Therefore, phenols can provide a unique flavor for Xuecai produced in JX.

Eleven aldehyde compounds were present in the Xuecai samples. They contributed 9 and 7% of the total volatiles content in JX and NB samples, respectively. Benzaldehyde (L5) was the major aldehyde in JX (49.80 μg/kg) and NB (38.97 μg/kg) samples. This compound accounted for 46 and 62% of the total aldehyde content in JX and NB samples, respectively. According to Zhao et al. (2007), benzaldehyde cannot be detected in fresh potherb mustard but can be detected in fermented potherb mustard. This indicates that benzaldehyde is mainly produced by microbial metabolism, and it can provide Xuecai with a pleasant almond flavor.

Although eight ketone compounds were detected in the Xuecai, they contributed only 3 and 2% of the total volatiles content in JX and NB samples, respectively. Aromatic-turmerone (K6) and β-ionone (K5) were the most abundant ketones in JX and NB samples, respectively. These ketone compounds can not only provide some fruity odors to the Xuecai but also have probiotic functions. For example, aromatic-turmerone has been shown to display anti-angiogenic activities and β-ionone displayed chemopreventive activity against hepatocarcinogenesis (Yue et al., 2015; Miranda et al., 2019). Thus, Xuecai could potentially be a functional fermented food.

For the alkene compounds, α-curcumene (AL2), α-zingiberene (AL3), β-bisabolene (AL4), and β-sesquiphellandrene (AL5) were only detected in JX samples, and in combination they contributed 5% of the total volatiles content. 3,5,5-Trimethyl-2-hexene (AL1) was only detected in NB samples and contributed 0.2% of the total volatiles content. These differences in the types of alkene compounds between JX and NB samples may be related to differences in the bacterial communities and raw materials. This is because the lipid from raw materials can be converted into alkenes *via* microbial metabolic pathways (Chen et al., 2015).

In addition, two acids and seven others were identified in the Xuecai samples. Their combined contribution to the total volatiles content was less than 5% in JX and NB samples, respectively. Among them, dimethyl disulfide (O1) and dimethyl trisulfide (O4) were only identified in NB samples. These compounds are major volatile sulfur components in cooked cabbage and need high temperatures to form. Therefore, it is possible that the temperature of the fermentation was higher in NB than in JX.

### Comparative Analysis of the Main Flavor Compounds

OPLS-DA was applied in this study to examine the main flavor compounds in the two types of Xuecai. The first two principal components accounted for 76.6% of the variation. The score scatter plot showed a trend of separation according to the Xuecai fermentation technology (Figure 1B). To identify the flavor compounds responsible for the separation, variable importance for projection (VIP) and S-plots were used. A VIP value greater than 1 is typically used to indicate a significant contribution to the model. A total of eight volatile compounds were found to have a VIP score > 1: E9, P2, N1, E1, E14, L11, P1, and A1 (Figure 1C). Among them, only E9, N1, E1, L11, P1, and A1 exhibited *P* [corr(1)] > 0.5 and *P*(1) > 0.075 according to the S-plots (Figure 1D and Supplementary Table 3). Thus, it could be deduced that the contents of these six flavor compounds significantly differed between traditional fermentation Xuecai and modern fermentation Xuecai. N1 and E9 were found in higher amounts in NB than in JX samples. On the contrary, JX samples contained higher amounts of E14, ethanol (A1), phenol (P1), ethyl acetate (E1), and 3-(2,6,6-trimethyl-1-cyclohexen-1-yl) acrylaldehyde (L11). The differences in the contents of these flavor compounds could mainly be due to differences in the bacterial community. Thus, the bacterial community compositions of the Xuecai were analyzed using HTS.

### Comparative Analysis of Bacterial Communities

**FIGURE 2:**
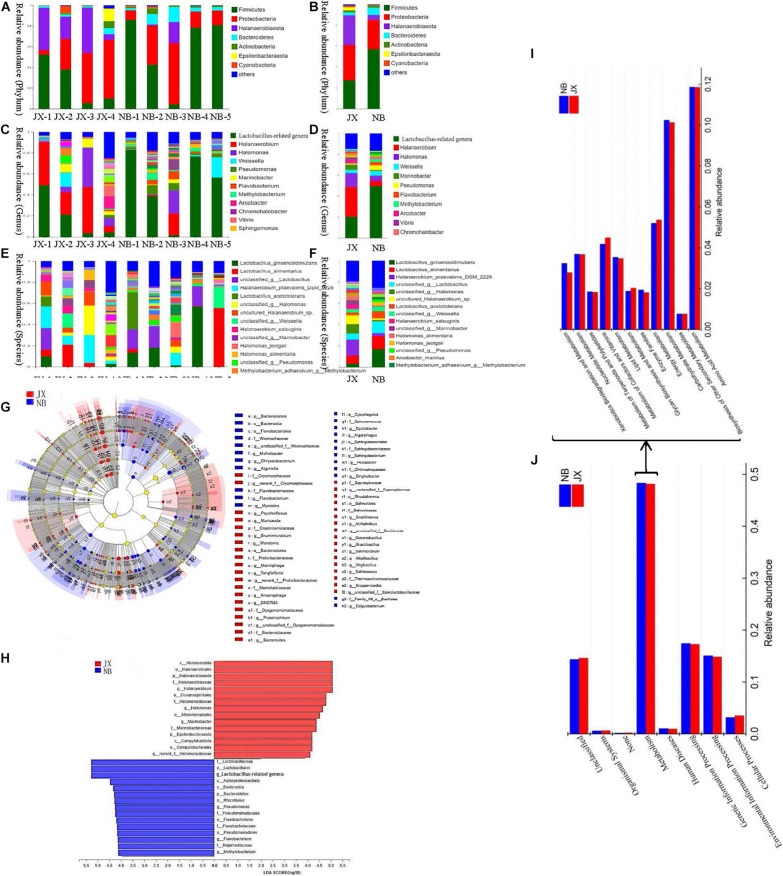
Composition, LEfSe comparison, LDA score and the predictive functions of bacterial community between traditional fermentation Xuecai (JX) and modern fermentation Xuecai (NB). **(A,B)** Composition of bacterial community at phylum level; **(C,D)** composition of bacterial community at genus level; **(E,F)** composition of bacterial community at species level; **(G,H)** LEfSe comparison and LDA score; **(I,J)**: the relative abundance of KEGG pathways (level 1) and the relative abundance of pathways in metabolism (level 2).

The numbers of effective sequences in the JX and NB samples were 51,685 and 49,875, respectively. Based on a 97% similarity threshold, 2509 operational taxonomic units (OTUs) and 2333 OTUs were obtained from the JX and NB samples, respectively. Of these, 1625 OTUs were shared among the different samples. Specifically, 884 and 708 OTUs were uniquely identified in the JX and NB samples, respectively (Supplementary Figure 3). The differences between the bacterial community profiles are shown in Figures 2A–F. A total of 39 phyla, 1147 genera, and 1995 species were identified in the JX and NB samples (Supplementary Table 4).

At the phylum level, Firmicutes, Proteobacteria, Halanaerobiaeota, and Bacteroidetes were the four dominant phyla in both JX and NB samples. The relative abundances of the four phyla in the NB and JX samples were 58.34, 27.18, 4.26, and 6.81%, and 25.19, 34.66, 29.09, and 2.77%, respectively. These four phyla have also been reported to be the main constituents of the bacterial flora in other Chinese fermented vegetables, such as pickled radishes and fermented tuber mustards (Liu and Tong, 2017; Rao et al., 2020).

At the genus level, the predominant genera in the JX samples were *Halanaerobium* (29.06%), *Lactobacillus*-related genera (18.31%), *Halomonas* (12.96%), and *Marinobacter* (4.96%). However, the major genus in the NB samples was *Lactobacillus*-related genera, which accounted for 51% of the relative abundance. *Lactobacillus*-related genera was followed by *Halomonas* (4.57%), *Halanaerobium* (4.25%), *Pseudomonas* (4.50%), and *Weissella* (4.33%) in the NB samples. *Halanaerobium praevalens* DSM 2228, unclassified_genus *Halomonas*, unclassified *Halanaerobium*. sp., *Lactobacillus alimentarius*, and unclassified_genus *Lactobacillus* were the five dominant species in the JX samples, accounting for 15.25, 8.69, 8.39, 7.74, and 4.58% of the relative abundance, respectively. However, the predominant species in the NB samples were *Lactobacillus ginsenosidimutans* (18.27%), *L. alimentarius* (11.25%), unclassified_genus *Lactobacillus* (10.95%) and *Lactobacillus acetotolerans* (6.93%). Furthermore, the LEfSe algorithm (LDA log score threshold ≥4) was used to reveal significant differences in bacterial structures between the JX and NB samples (Figures 2G,H). JX Xuecai samples had greater proportions of *Halanaerobium*, *Halomonas*, and *Marinobacter*, whereas NB samples had greater relative proportions of *Lactobacillus*-related genera and *Pseudomonas*.

The TA and NaCl concentration have been shown to be two major factors regulating bacterial community in fermented vegetables (Lee et al., 2017; Zhao et al., 2020). However, the regulation of bacterial community by NaCl occurs mainly in the early fermentation stage (Guan et al., 2020; Zhao et al., 2020). In the present study, the Xuecai samples were in the late fermentation stage. TA has been shown to be the dominant factor regulating bacterial community at the later stage (Zhao et al., 2020). According to a recent report, the abundance of *Lactobacillus*-related genera increased with increasing TA (Zhao et al., 2020). The results of the present study showed that the TA was lower in modern fermentation Xuecai (NB) samples than in traditional fermentation Xuecai (JX) samples (Table 1), but the abundance of *Lactobacillus*-related genera was significantly greater in NB than in JX samples. This result is contrary to previously reported. This can mainly be explained by the different curing environments. Traditional fermentation technology (JX) involves wilting the raw materials in the sun, removing the brine by inversion, and sealing the fermentation vessels with mud. Therefore, the curing environment used in JX has less water and oxygen than that used in NB.

Some halophilic bacteria (HB), such as *Halanaerobium*, can grow better in an anaerobic and dry environment, and inhibit the growth of *Lactobacillus*-related genera. Therefore, the oxygen and water contents may play specific roles in regulating the bacterial community composition during fermentation. Genera of LAB, especially *Lactobacillus*-related genera, have been reported to be the most dominant genera in Chinese fermented vegetables (Zhang et al., 2018). However, interestingly, in the present study, the bacterial community of the JX samples was mainly composed of HB (with a total abundance of 47%), such as *Halanaerobium*, *Halomonas*, and *Marinobacter*. This finding is consistent with our previous research (Zhang et al., 2019). HB as the principal bacteria present in Chinese Xuecai. Thus, these bacterial community differences are responsible for the flavor differences between JX and NB Xuecai.

### Prediction of a Preliminary Flavor Formation Mechanism

**FIGURE 3:**
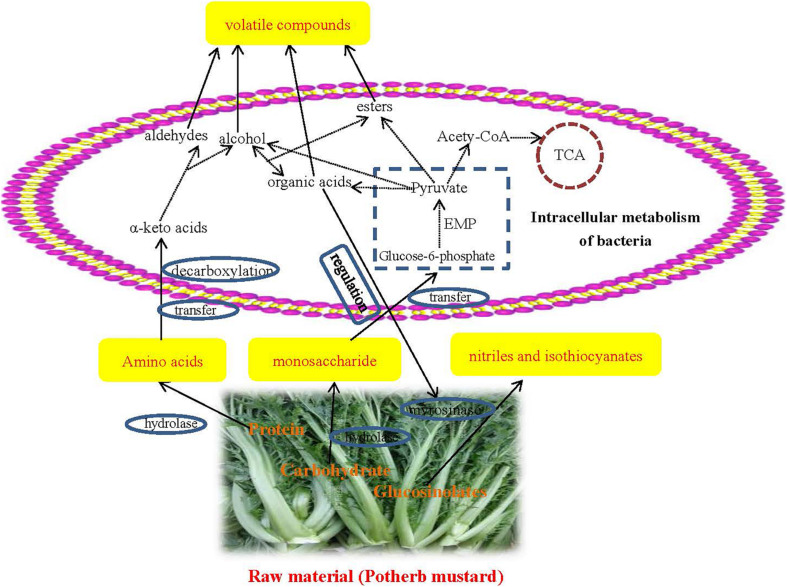
Network diagram of preliminary formation mechanism of volatile flavors during the fermentation of Xuecai.

Phylogenetic Investigation of Communities by Reconstruction of Unobserved States was used to predict the function of the bacterial community. The results of the KEGG pathway analysis showed that the proportion of metabolism-related genes was the largest in JX and NB samples (level 1), indicating that metabolism was the most important function of the bacterial community. In particular, the abundance of OTUs related to carbohydrate metabolism and amino acid metabolism was significantly higher than the abundance of OTUs related to other metabolic pathways (Figures 2I,J and Supplementary Table 5). This indicated that proteins and carbohydrates were the main materials responsible for flavor formation. The network pathway for the formation of volatile flavor compounds is shown in Figure 3.

At first, proteins and carbohydrates from the potherb mustard are degraded to amino acids and monosaccharides *via* hydrolases. These monosaccharides are transported into the cell and are utilized by glycolysis, producing pyruvate. Pyruvate is the central compound for organic acid, alcohol, and ester formation. In addition, amino acids are converted to α-keto acids by aminotransferases. Alpha-keto acids are subsequently decarboxylated to synthesize alcohol and aldehyde compounds. Alcohol compounds and organic acids can be used to synthesize esters *via* esterification reactions performed through the action of microorganisms. These secondary metabolic pathways were also successfully predicted at KEGG pathway level 3. For example, glycolysis/gluconeogenesis (ko 00010), amino acid related enzymes (BR: ko01007), pyruvate metabolism (ko00620), and arginine and proline metabolism (ko00330) were very enriched in JX and NB samples (their abundances were in the top 20; Supplementary Table 5).

Glucosinolates from *Brassicaceae* plants can be metabolized by myrosinases to generate nitriles and isothiocyanates. However, the activity of myrosinases is determined by environmental acidity. For example, nitriles are major degradation products at pH 2–4, and isothiocyanates are produced at pH 4–5 (Gil and Macleod, 1980). Thus, some acid-producing bacteria, such as *Lactobacillus*-related genera, and *Halomonas*, can affect the formation of nitriles and isothiocyanates. However, it was not possible to obtain a detailed formation mechanism for the flavor compounds under the action of bacteria and enzymes. A metagenomic and culture-dependent analysis will be used to reveal the detailed mechanism for the role of specific bacteria in flavor compound formation in our future research.

### Correlations Between Bacterial Community and Flavor Compounds

**FIGURE 4:**
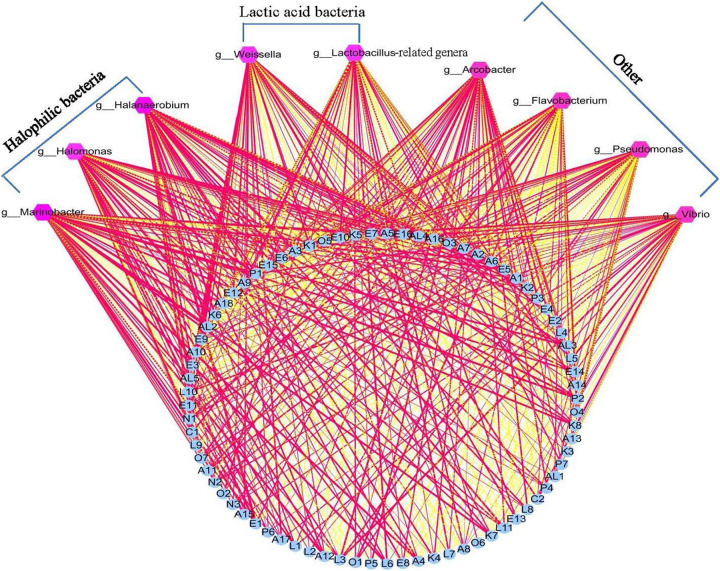
Visualization of the correlation network according to significant correlations between flavor compounds and the relative abundance of the bacteria. Each blue roundness represents a flavor compound, each pink polygon represents a genus. The red solid line and yellow dotted line represents positive and negative correlation, respectively. In addition, line width indicates the strength of correlation. For interpretation of the code in this figure, the reader is referred to the Table 2 and Supplementary Table 2.

Lactic acid bacteria have been reported to produce primary metabolites such as glucose, amino acids, and fatty acids, using raw vegetable materials. These primary metabolites are further decomposed into volatile secondary metabolites *via* LAB metabolism. Previous reports have thus suggested that LAB play an important role in the formation of the flavor of fermented vegetables (Xiao et al., 2020). However, in the present study, the correlation analysis results showed that there were no significant relationships between LAB (*Lactobacillus*-related genera) and flavor compounds in the Xuecai samples (Figure 4 and Supplementary Table 6). Instead, some HB (*Halanaerobium*, *Halomonas*, and *Marinobacter*) exhibited a high positive correlation (|*r*| > 0.7 and *P* < 0.05) with flavor substances such as E1, E3, E5, L6, L11, A4, A6, A12, K6, K7, K8, AL3, AL4, AL5, P1, and P2. This is a novel insight that HB provides an important contribution to the flavor formation of Xuecai.

However, in the present research, the bacterial community and flavor compounds were only analyzed at the late fermentation stages. The dynamic changes in bacterial community and flavor compounds that occur in different fermentation stages are unclear. LAB may be an important contributor to flavor formation during the early or middle fermentation stages. The formation of some flavor compounds, such as isothiocyanate and nitrile compounds, is influenced by changing the acidity of the fermented vegetables. Thus, LAB can indirectly affect flavor formation through organic acid metabolism. This also explains why the content of isothiocyanates (E7, E9, E11, and E12) and nitriles (N1 and N2) in the NB samples were significantly higher than in the JX samples.

## Conclusion

This study provided a comparative analysis and formation mechanism for flavor compounds and bacterial communities in Xuecai produced by traditional and modern fermentation. HS-SPME GC-MS was used to identify volatile flavor compounds. A total of 77 volatile compounds were identified in the Xuecai. OPLS-DA analyses showed that among these compounds, there were significant differences in the contents of E9, N1, E1, L11, P1, and A1 between traditional and modern fermentation Xuecai. *Lactobacillus*-related genera was the most abundant genus (50%) in Xuecai produced by modern fermentation. However, traditionally fermented Xuecai was dominated by HB (*Halanaerobium* at 29.06% and *Halomonas* at 12.96%). No significant relationships were found between *Lactobacillus*-related genera and flavor compounds in the Xuecai. Nevertheless, *Halanaerobium*, *Halomonas*, and *Marinobacter* were found to be the main microorganisms related to the synthesis of flavor substances. KEGG pathway analysis showed that carbohydrate metabolism and amino acid metabolism were the most abundant pathways. This indicated that flavor compound formation was mainly dependent on protein and carbohydrate degradation. The current study provides novel insights into the use of bacterial resources from Xuecai, and HB is an important bacteria for flavor formation of Xuecai.

## Data Availability Statement

The original contributions presented in the study are publicly available. This data can be found here: NCBI, PRJNA683095.

## Author Contributions

JZ: experimental design, experiment, writing, and data analysis. CZ: funding acquisition and modify the article. XX: analysis of physicochemical properties and GCMS. DL: funding acquisition, experimental design, and supervision. WZ: sample collection. All authors contributed to the article and approved the submitted version.

## Conflict of Interest

WZ was employed by the company Hangzhou Trendbiotech Co., Ltd. The remaining authors declare that the research was conducted in the absence of any commercial or financial relationships that could be construed as a potential conflict of interest.

## Footnotes

1 http://huttenhower.sph.harvard.edu/galaxy
