# Supplementary material for: Comparative Analysis of Traditional and Modern Fermentation for Xuecai and Correlations Between Volatile Flavor Compounds and Bacterial Community
Source: Front Microbiol. 2021 Apr 29;12:631054. doi: 10.3389/fmicb.2021.631054 (PMC8118120; doi:10.3389/fmicb.2021.631054)
Supplement: Supplementary file 1 [file Data_Sheet_1.DOCX]

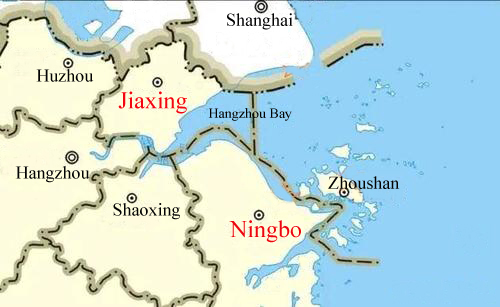

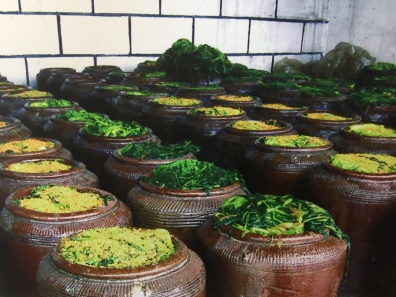

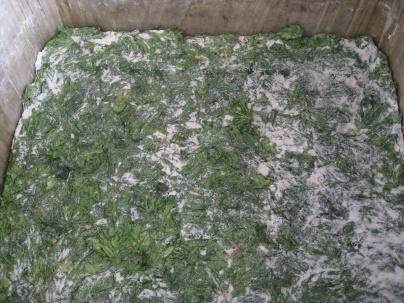


earthenware pots

cement containers

Supplementary Fig.S1. Collection locations and fermented container of Xuecai


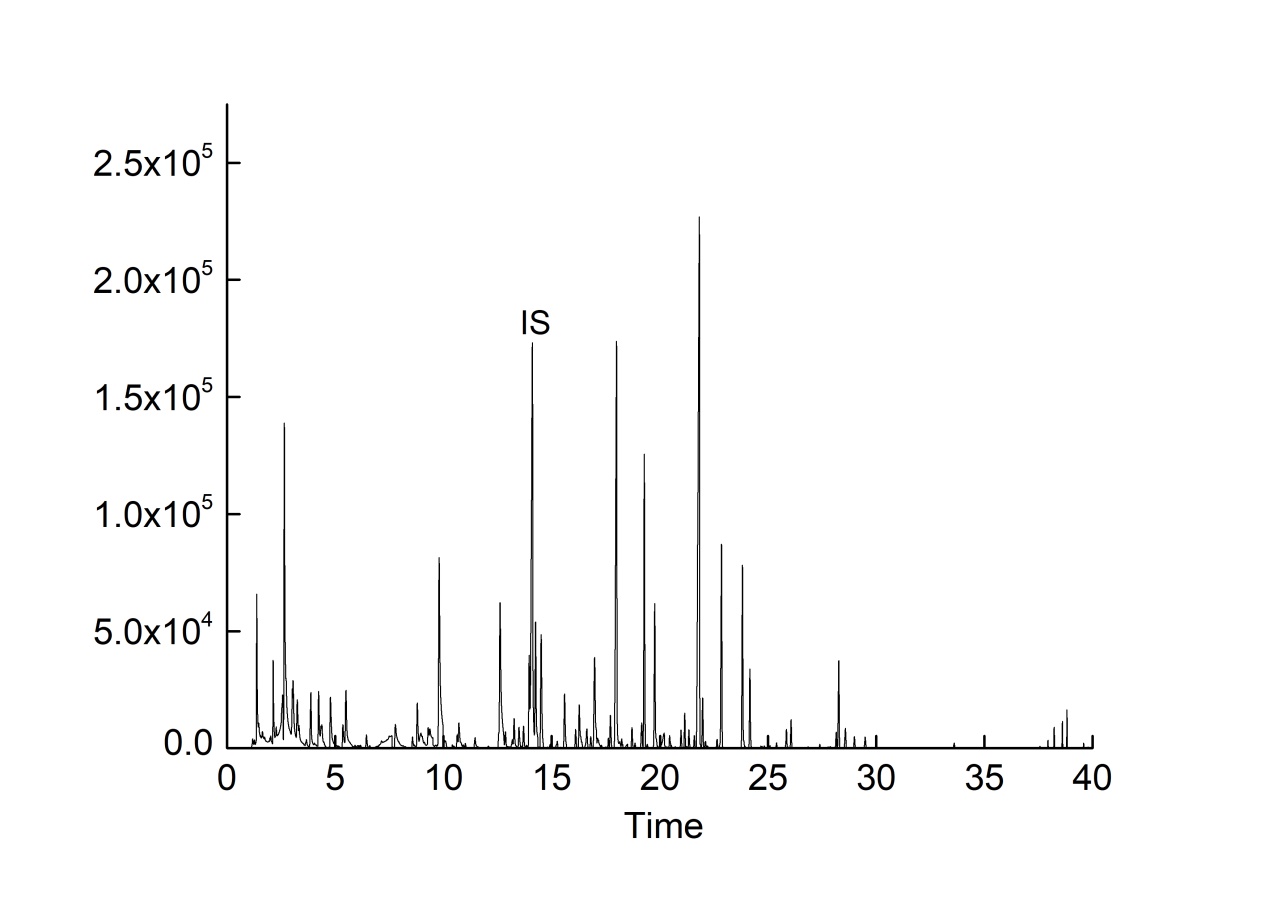


Supplementary Fig.S2. Representative GCMS chromatograms of volatiles in Xuecai [NB-3 with highest types (44) of volatiles]. IS represents the internal standard (3-octanol) used.


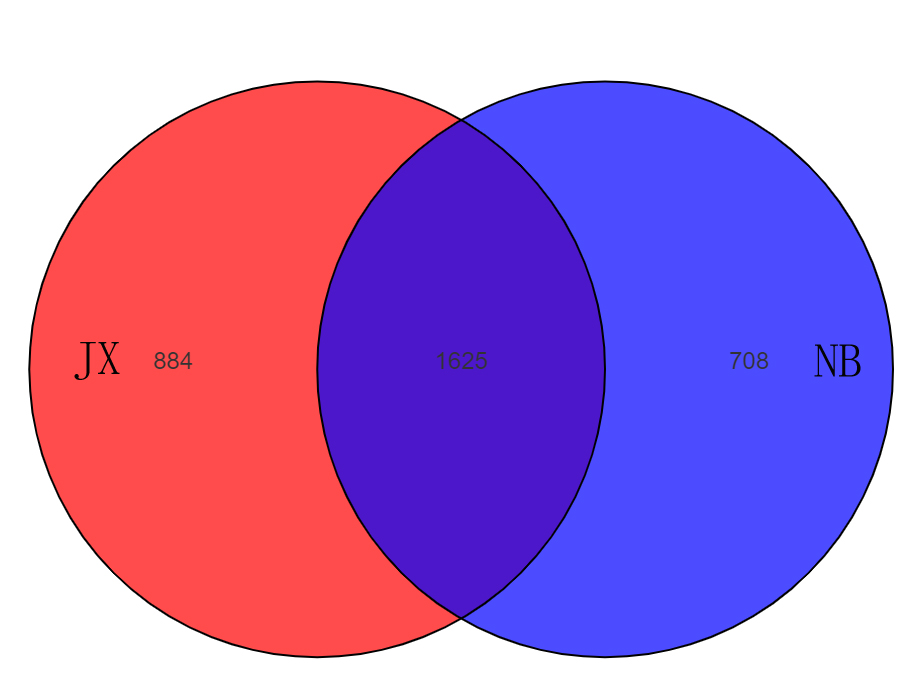


Supplementary Fig.S3. Venn plot for bacteria between traditional fermentation Xuecai (JX) and modern fermentation Xuecai (NB). The figures in different compartments mean the unique and shared OTUs.
